# Cognitive, emotional and expressive factors determining the quality and variability of mentalization styles

**DOI:** 10.1192/j.eurpsy.2022.1771

**Published:** 2022-09-01

**Authors:** A. Vologzhanina, E. Sokolova, A. Ryzhov, L. Pechnikova

**Affiliations:** Lomonosov MSU, Faculty Of Psychology, Moscow, Russian Federation

**Keywords:** mentalization, SCORS-G, psychological help, tolerance to ambiguity

## Abstract

**Introduction:**

In contemporary context the difficulties of making sense of social ambiguity becomes one of the most important appeals for seeking the psychological help. This grounds the importance of studying the mechanism underlying the quality of mentalization and its individual variations.

**Objectives:**

The objective of the study was to find empirical relations between the quality of mentalization and its cognitive, emotional and expressive mediating factors.

**Methods:**

(1) The Adult Attachment Interview, scored using Social Cognition and Object Relations-Global rating method for mentalization ability. (2) Group embedded figures test. (3) New Tolerance-Intolerance to ambiguity and (4) Toronto alexithymia scale questionnaires. Twenty participants, aged 18-38, looking for psychological consultation, took part in the study.

**Results:**

Correlation analysis suggests positive relation between field-independency and tolerance to ambiguity (r = .47; p < .05). The complexity of representations of the mind positively correlates with the understanding of social causality (r = .92; p < .01). The affective quality of relationships’ representations positively correlates with the ability to emotionally invest into relationships (r = .66; p < .01), and with the understanding of social causality (r = .47; p < .05). The ability of emotional investment into relationships also positively correlates with the understanding of social causality (r = .93; p < .01). There is a negative link between the severity of alexithymia and the presence of long-term relationships with a partner (r = -.53; p < .05).

**Conclusions:**

Mentalization should be understood as a system, with underplaying cognitive, expressive and emotional factors.

**Disclosure:**

No significant relationships.

